# The effects of introducing an electronic prescription system with no copayments

**DOI:** 10.1186/s13561-015-0056-4

**Published:** 2015-07-16

**Authors:** Ida Iren Eriksen, Hans Olav Melberg

**Affiliations:** 1 Institute for Health and Society, University of Oslo, Oslo, Norway; 2 University of Oslo, OCBE and Department of Health Management and Health Economics, Box 1089 Blindern, 0317 Oslo, Norway

**Keywords:** Electronic prescriptions, Financial incentives, Copayment, Policy reform

## Abstract

**Background:**

To examine the impact of introducing an electronic prescription system with no copayments on the number of prescriptions, the size of prescriptions, and the number of visits and phone calls to primary physicians.

**Methods:**

Fixed regression models using monthly data on per capita prescriptions claims and consultations between 2009 and 2013 at the municipality level, before and after the introduction of the electronic prescription system.

**Results:**

The electronic prescription system with no copayment increased the number of prescriptions by between 6.0 and 8.1 %. It decreased the average size of each prescription, but it did not decrease the number of consultations.

**Conclusion:**

The reduced direct and indirect costs of obtaining prescriptions after the introduction of the electronic prescription system changed the financial incentives facing the patients and physicians. This led to significant changes in the level and size of prescriptions and illustrates the importance of financial incentives.

## Background

A recurring theme in the literature on physicians’ behaviour is the extent to which physicians are influenced by changes in incentives as opposed to pure medical considerations [1–3]. This article contributes to a subfield of this debate by examining how patterns of prescription behaviour are affected by the introduction of an electronic prescription system, which changed the incentives facing the physicians and the patients. These effects are often difficult to analyse because usually the systems are adopted either voluntarily and gradually or mandatory for all at a single point in time. With voluntary adoption the effects cannot be generalised and when all adopt the change simultaneously it is difficult to control for other factors that may change at the same time. Using data from a gradual, but mandatory, introduction of an e-prescription system in Norwegian municipalities starting from 2011, it is possible to reduce these problems. The effect of electronic prescription systems and associated reimbursement rates is of general interest given that many countries are still considering how and when to implement e-prescription systems [4, 5].

The existing literature on electronic prescription systems has often focused on consequences in terms of potentially reducing prescription errors [6–8], the practical obstacles and experiences of physicians [9], and the experience of patients [10]. For instance, one study reported a 50 % reduction in the daily time spent on refills with an electronic prescription system [11]. Reduction in time, errors and changes in user experience are important aspects of e-prescription reforms, but the Norwegian experience also makes it possible to examine how the introduction of electronic prescription affects the total number of prescriptions. An electronic prescription system will change several of the incentives facing the physicians and the patients through the direct and indirect costs associated with prescriptions. Indirectly it will change costs by reducing the time required to get and deliver a prescription. Directly, it will change the costs since a new system is often accompanied by a new fee structure, with different total payments and changes in the co-payment rates. This paper estimates the overall net effect of the new incentives facing the physicians and the patients after the introduction of a policy reform involving electronic prescriptions and its new fee structure.

The main hypothesis is that the reform increased the overall number of prescriptions because the new e-prescription system reduced the direct and indirect costs of a prescription for both the patient and the physician. Before the system was introduced, the direct financial cost for the patient obtaining a prescription without a consultation was about 5 Euro while the state paid 1 Euro. After the electronic prescription system was introduced in a municipality, the co-payment for obtaining a prescription was eliminated. Instead the state reimbursed the physician a fixed sum of about 6 Euro for each prescription they administered. In addition to reducing the financial cost to the patient, the indirect costs associated with the prescription were reduced since the patient no longer had to get the paper prescription physically. This meant that they no longer had to visit the office in person, nor did the physician have to fax the prescriptions to the pharmacist. The elimination of the co-payment would also increase the number of prescriptions if physicians were less conscious of costs reimbursed by the authorities as opposed to co-payments paid by patients.

The second hypothesis is that the physicians decreased the size of each prescription when the transaction costs associated with prescriptions were reduced. It was financially profitable to split large prescriptions into smaller and more frequent ones because the physicians were paid per prescription. In the new system more frequent prescriptions were also easier to justify since the costs to the patient decreased. Previous research indicates that the amount of co-payment affects physicians’ prescription behaviour [12–14]. More frequent prescriptions with smaller amounts are also considered safer in terms of reducing accidents and overdoses.

Thirdly, the electronic prescription system was expected to reduce the number of consultations, both in person and over the phone. After the reform, it was in many cases possible to obtain a prescription by using forms on the web and email i.e. without directly consulting the physician. The physician would receive a list of requests electronically and as long as it was deemed medically acceptable to give the prescription without further investigations, they only had to click a button to send the prescriptions. Less time spent on the phone or in the office giving prescriptions could free up time, and for this reason it was expected that the number of consultations would drop slightly when electronic prescriptions was introduced.

Norwegian General Practitioners (GPs) work mainly in privately owned practices with contractual agreements with the municipality in which they work to provide medical services [15]. Patients can choose their GP and can change GP twice a year, which means GPs have to compete with each other for patients. They are financed partly through a fee-for-service (about 75 % of total income) and partly through a fixed reimbursement per person they are responsible for. The fee-for-service payment is administered through the Electronic Patient Record (EPR), where the doctor registers an invoice for every refundable service. The invoice contains a code to categorise the type of service provided. All the invoices are transferred to The Norwegian Health Economics Administration (HELFO), who is responsible for reimbursing the doctors according to the fee-for-service scheme. Since 2005 the invoices have been saved in the KHUR-register owned by HELFO and our analysis is based on data obtained from this registry. The data contains information on the monthly number of claims for different types of reimbursements from all the physicians in the 428 municipalities in Norway.

The exact amount of the fee for the different services is negotiated by The Norwegian Medical Association, The Norwegian Association of Local and Regional Authorities (KS) and the government, and published in the standard tariff. They decide both the total amount for each tariff and the share of the state reimbursement vs. the patients’ out of pocket payment. Table 1 shows the total reimbursement rates to the physician for administering a regular paper-based prescription and an electronic prescription in the different years, as well as how much of the total that was paid by the patient and the state.

**Table 1:** Total reimbursement rates to physicians for providing paper based vs. electronic prescriptions and the amount paid by the state vs. the patient

	Paper based prescriptions (NOK)			Electronic prescriptions (NOK)		
	Total	State	Patient	Total	State	Patient
2008-2009	50	15	35			
2009-2010	45	5	40			
2010- 2011	50	5	45			
2011-2012	50	5	45	50	50	0
2012-2013	54	9	45	54	54	0
2013-2014	55	9	46	55	55	0

The e-prescription system was introduced gradually. The logic behind the introduction was that more densely populated areas and cities should implement the system before less densely populated areas around the city-centres. In that way it would be easier for the areas around the city-centres to start using e-prescriptions since the cities around them already had the infrastructure implemented and tested. When the government introduced e-prescriptions for GPs it was decided that patients should no longer pay for prescriptions written outside of doctor visits. Physicians could write prescriptions to patients without seeing them in a consultation if the patient and the condition the prescription is requested for are unproblematic and well known to the doctor. A typical situation is the renewal of pharmaceuticals for chronic diseases. In this case the patient calls the doctor’s office with a request, the prescription is prepared, often by the administrative staff, signed by the doctor, and the patient picks it up in the doctor’s office. When the prescription was sent by post or fax, the patient also had to pay an additional administration fee of about 7 Euro (54 NOK). Through the years, the proportion of the state reimbursement vs. the patients’ out of pocket payment for this service has varied, but the patient has had to pay approximately 80 % of the cost of paper prescriptions written outside of consultations. The same service is provided also after the introduction of e-prescriptions, but the patient no longer has to pay the administration fee.

## Method

**Fig. 1:**
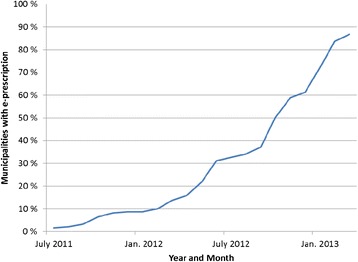
Percentage of all municipalities with electronic prescription systems

We use data from monthly reimbursement claims between 2009 and 2013 to study the effect of the change in the tariff made after the introduction of electronic prescriptions in Norway. The system of electronic prescription was introduced gradually in different municipalities starting in 2011 (see Fig. 1).

Given the existence of many observations for different municipalities across time, we used a fixed effects model to study the effect of the electronic prescription system [16]. In the model, the dependent variable is the number of reimbursements per capita (*y*) in each municipality (*i*) for every month in the period between 2009 and February 2013 (*t*). The number of reimbursement claims is modelled using a fixed effect for each municipality (*α*_*i*_), a dummy for the introduction of the electronic prescription system in the municipalities (*D*_*i*,*t*_) and eleven monthly dummies (*M*_*j*_), using January as a reference month. The impact of the prescription system is estimated by the coefficient *δ* and the effect of the month of the year on prescriptions is estimated by *β*_*j*_ where *j* is the month of the year. Summarising this, the model is given by the following equation, which is discussed in more detail below:

$$ {y}_{i,t} = {\alpha}_i+\delta\ {D}_{i,t}+{\displaystyle \sum_{j=2}^{j=12}}{\beta}_j{M}_j+{\varepsilon}_{i,t} $$

The number of claims in a municipality may vary for many reasons, and using a fixed effects model means that the time-invariant variables that are specific to the municipality are captured by a separate term for each municipality [17]. For instance; municipalities with an older population may have a stable and higher prescription rate than municipalities with a younger population. Similarly, prescription patterns in urban municipalities may be different than the prescriptions patterns in rural areas. These types of fixed factors are captured by the municipal fixed effects in the regression.

In addition to capturing municipal differences, the fixed effect term also reduces a potential measurement problem in the dependent variable. The reimbursement code used for claims related to paper prescriptions is sometimes also used for other services (long-term sickness leave certificates and requisitions for physical therapy). In contrast, the reimbursement code for electronic prescriptions is used only for prescriptions. The dependent variable in the model is the total number of claims in both the category for electronic and paper based prescriptions. This aggregate variable includes some non-prescription based claims that cannot be eliminated since they are reimbursed using the same code as prescription claims. This means that the dependent variable could differ between municipalities even if the number of prescriptions is the same. However, in the fixed effect model the time-invariant municipal differences in the level of the other claims are eliminated. For instance, a municipality with a higher than average level of long-term sick leaves would have a higher level of claims, but as long as the high sick leave pattern is a trait of the municipality it does not affect the analysis of change associated with the electronic prescription system in a fixed effect model. In general, the existence of other influences does not create a bias as long as they do not systematically increase or decrease at the same time when the electronic system for prescriptions is introduced.

Finally the models include two categories of dummy variables. First, prescription patterns depend on disease patterns, which have strong and stable seasonal variations. In order to capture this, the model includes monthly dummy variables. Second, and most importantly, the model includes a dummy for the introduction of the electronic prescription system. The precise date for the implementation is based on the month when the number of claims was above a threshold. This threshold was initially defined as the first month when the municipality reported at least one reimbursement claim for electronic prescriptions. However, in practice the system took some weeks to be fully implemented in the municipalities and often one would observe less than ten electronic prescriptions in the first month of use. Given that one is interested in the effect of the system after it had been implemented, and not during the first implementation month when it may have been used only for a few days or by a few physicians, we set the dummy for e-prescription to be one when the municipality reported at least 50 reimbursement claims for electronic prescriptions. We also tested whether smaller thresholds (“at least one claim”) or higher thresholds (“at least 100 claims”) changed the results.

To test different aspects of the model we also ran several additional regressions. We tested if the effect of the reform differed depending on whether the municipality was an early or a late adopter of the reform, whether the effect became larger the longer the reform had been in place, and whether the effects also were present after introducing annual dummies. Finally, the method itself was tested using a simulation in which the actual dates of implementing the e-prescription system was replaced by random draws of assumed interventions at different dates. In order to believe the method, the results using the actual dates for the intervention should be different from the results under the assumption of interventions at random dates.

## Results

**Fig. 2:**
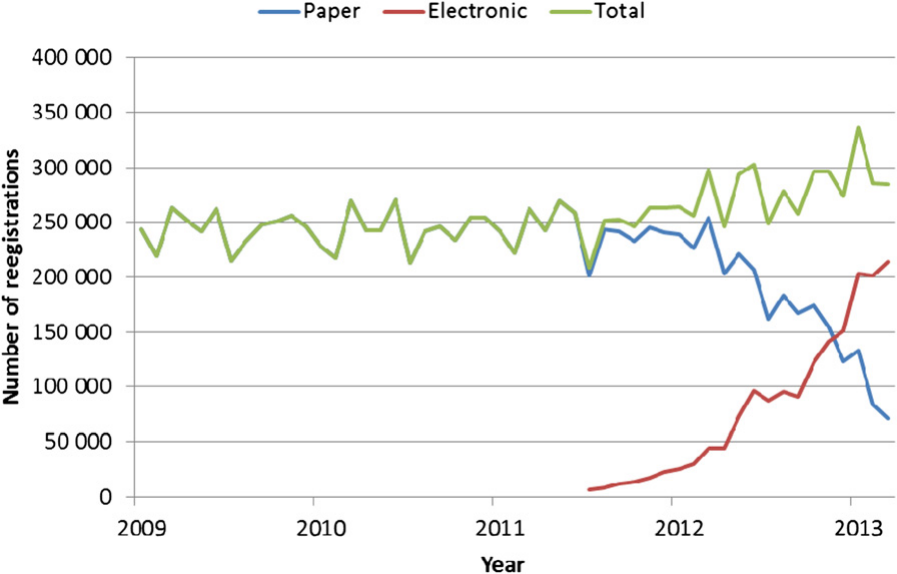
Monthly numbers of reimbursed claims at the national level (total, regular paper based and electronic prescriptions)

Figure 2 shows the growth in the number of electronic prescriptions, regular reimbursement claims and the overall number of reimbursements before and after the introduction of an electronic prescription on a national basis. Overall more than 17.5 million codes were registered from 2008–2012. The number of electronic prescriptions has experienced a steady increase and in 2012 more than 1 million electronic prescriptions were registered.

**Fig. 3:**
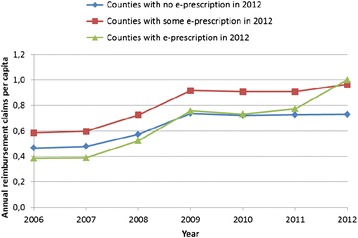
Number of reimbursement claims per capita in counties who introduced electronic prescriptions in 2012 (fully or partially) and counties that did not

The average number of prescriptions per capita per month in Norwegian municipalities between January 2008 and March 2013 was 0.08. This is equivalent to about one prescription per year for every person. Figure 3 shows the different trends in the number of total reimbursement claims per capita for counties that introduced the e-prescription in 2012 (fully or partially) and those counties that did not introduce e-prescriptions in 2012.

The numerical results from the fixed effect regression model of factors affecting the number of prescriptions are shown in Table 2. Based on the results from this regression, the introduction of an electronic prescription system is estimated to have increased the overall number of prescriptions by between 6.1 and 8.0 % depending on the threshold for when the system was considered to be fully implemented. The coefficient for the introduction of an electronic prescription system is statistically significant at the 5 % level in all regressions.

**Table 2:** Results from the fixed effect regression model estimating how the electronic system and other factors affected the number prescriptions (per capita per month in municipalities, N = 21 419)

	Threshold: 1		Threshold: 50		Threshold: 100	
E-prescription (Dummy)	0,0060	***	0,0049	***	0,0046	***
February	−0,0065	***	−0,0064	***	−0,0064	***
March	0,0028	***	0,0020	***	0,0030	***
April	−0,0035	**	−0,0039	***	−0,0040	***
May	0,0013	**	0,0010	*	0,0008	
June	0,0039	***	0,0026	***	0,0036	***
July	−0,0089	***	−0,0090	***	−0,0090	***
August	−0,0022	***	−0,0023	***	−0,0023	***
September	−0,0030	***	−0,0030	***	−0,0030	***
October	−0,0009	*	−0,0010	*	−0,0009	*
November	0,0018	**	0,0018	**	0,0018	***
December	0,0003		0,0005		0,0005	
Constant term	0,0751	***	0,0758	***	0,0760	***

*** Statistically significant at the 1 % level (**; 5 %, *; 10 %). The different thresholds represent how many times e-prescriptions have to be used in a month before the system is considered implemented in the municipality. Robust estimation of standard errors was used since the Modified Wald test indicated presence of groupwise heteroscadasticity

**Fig. 4:**
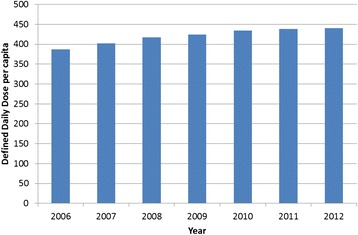
Pharmacautial market, Defined Daily Dose per capita from 2006 to 2012

Figure 4 shows the number of standardised daily doses of pharmaceuticals sold in Norway between 2006 and 2012. Despite the increase in the number of prescriptions, there was no equally large increase in the amount of total pharmaceuticals consumed. Between January 2012 and 2013 the share of municipalities with an electronic prescription system increased from 9 to 72 %. At the same time the number of reimbursement claims for the code used for reimbursing prescriptions increased by 9.6 %, while the amount of pharmaceuticals sold measured in daily does per capita increased by 0.5 %. Taken together the increase in the number of prescriptions and the stable statistics for daily dosage, indicate a decline in the amount of pharmaceuticals prescribed in each prescription.

Table 3 shows the regression result for the number of reimbursement for office-based visits to the physician and phone consultations (not resulting in prescriptions). There is no evidence of a significant decline either in the number of office-based consultations or the number of phone consultations after the introduction of an electronic prescription system. Instead there seems to have been a very small, but statistically significant, increase in the number of phone consultations. There is no statistically significant change on the number of office consultations associated with the introduction of the electronic prescription system.

**Table 3:** Fixed effect regression of the per capita monthly number of daytime phone consultations with patients and daytime office consultations

	Daytime phone consultations			Daytime office consultations		
Variable	Coefficient		Standard Error	Coefficient		Standard Error
E-prescription	0,0028	***	0,0009	−0,0005		0,0013
February	−0,0058	***	0,0002	−0,0255	***	0,0009
March	−0,0008	**	0,0004	−0,0058	***	0,0008
April	−0,0127	***	0,0005	−0,0638	***	0,0028
May	−0,0064	***	0,0005	−0,0194	***	0,0010
June	−0,0053	***	0,0005	−0,0154	***	0,0011
July	−0,0266	***	0,0008	−0,0781	***	0,0040
August	−0,0140	***	0,0007	−0,0287	***	0,0017
September	−0,0066	***	0,0007	−0,0014		0,0020
October	−0,0031	***	0,0062	0,0029	*	0,0016
November	0,0017	***	0,0000	0,0139	***	0,0016
December	−0,0128	***	0,0006	−0,0308	***	0,0020
Constant term	0,0773	***	0,0004	0,2532	***	0,0011

*** Statistically significant at the 1 % level (**; 5 %, *; 10 %). Robust estimation of standard errors was used since the Modified Wald test indicated presence of groupwise heteroscedasticity

Table 4 shows the key results when the model was extended. The first extension introduced a dummy for late adopter which was defined as the municipalities which introduced the reform after half of the others had done so. The coefficient for municipalities who adopted the reforms later than the others (0,0009) was small and not statistically significant, while the coefficient for the reform (0,0047) remained significant. The second model allowed the effect to grow over time by introducing a linear trend which increased by one unit every month after the reform was introduced. The results indicate that the reform itself was still significant (ie. creating a shift of 0,0008), but also that the effect grew over time as people adapted and learned about the new possibilities (with a the coefficient of 0,0006 for every month the reform had been in place). In the third model with annual dummies, the effect of the reform was reduced (0,0008), but remained statistically significant. The annual dummies are correlated with the introduction of the reform since this happened mainly in 2012 and 2013 and the dummies are also large and significant for these years (0,0037 and 0,0087).

**Table 4:** Key results from regressions testing whether the effect differed depending on the time of intervention, the length of time after the intervention, and whether annual dummies would eliminate the effect (N = 21 419)

	Time of intervention adopters		Time after intervention		Annual dummies	
E-prescription (Dummy)	0,0047	***	0,0008	*	0,0008 ( (0,0004)	**
Dummy for late adopters	0,0009					
Linearly increasing effect			0.0006	***		
Dummy for 2010					−0,0007	**
Dummy for 2011					0,0006	*
Dummy for 2012					0,0037	***
Dummy for 2013					0,0087	***

*** Statistically significant at the 1 % level (**; 5 %, *; 10 %)

**Fig. 5:**
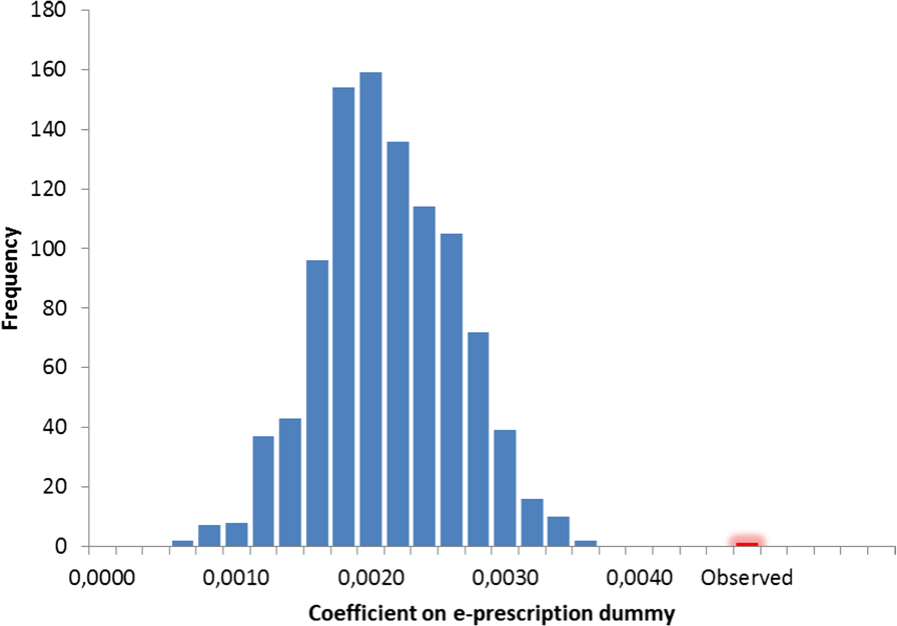
Distribution of coefficients for effect of electronic precriptions in one thousand regression simulations assuming random dates for the intervention (observed result with true dates marked in red)

The results from the thousand regressions each using randomly drawn dates for the implementation of the electronic prescription system in each municipality, is presented in Fig. 5. The coefficients in these regressions ranged from 0.0005 to 0.0036, compared to the regression coefficient of 0.0049 in the main analysis when using the true time period of implementation (threshold 50, Table 2).

## Discussion

In line with the first and second hypotheses, the results indicate that the new system increased the number of prescriptions and reduced the average size of each prescription. The third hypothesis, however, was rejected: The electronic system did not lead to a decrease in the number of office or phone consultations. There are several reasons as to why this did not occur. First of all, the new system required physicians to contact individuals asking for an electronic prescription when there were doubts about the appropriateness of the prescription. This could lead to more phone consultations, instead of the reduction that some policy makers expected. Second, physicians are in high demand and whatever time is freed up by a new technology is quickly filled with other tasks and patients.

The results strengthen the small but important literature on the effect of co-payment on physician behaviour. Several previous results have shown that physicians believe it is important to consider patients’ costs [18, 19], and that they also act on this belief in practice [20]. In the Norwegian case, there was no change in the overall reimbursement to the physician for each prescription, but the cost born by the patient decreased. The change towards more frequent prescriptions is consistent with the view that physicians pay less attention to the costs when they are born by the state and not the patient. Since Norwegian GPs are competing for patients, one reason for why the number of prescriptions has gone up might be that it becomes easier for the GPs to offer more prescriptions. More prescriptions to the patients might be an advantage in holding on to patients.

The reform had a statistically significant effect on prescriptions in in the extended models, even after the inclusion of annual dummies that are partly correlated with the reform itself. These extensions also revealed that there was a learning effect: Over time providers adapted by creating easier web based solutions for patients, and patients also learned about the lower copayments over time.

While the results show that the number of prescriptions went up, there are several potential limitations and issues for further studies in the field. First of all, although a fixed-effects model captures time-invariant factors, it is vulnerable to the effects of time-varying covariates. For instance, if the reform had been introduced during a recession one might observe a decrease in the number of prescriptions after the reform, but the reduction might be more related to the economic change than the reform itself. Failing to control for income would then lead to the misleading conclusion that the reform decreased the number of prescriptions. This is a general limitation with fixed-effect models, but in our specific case the problem is reduced because the reform was introduced at different points in time in different municipalities. It is unlikely that income and other factors would suddenly fall at different points in time in different municipalities exactly when the electronic prescription system was introduced. Also, the results from the simulation show that the results cannot be explained by a general trend over the whole time period since the regression results from using the true dates was much larger than all the results from the simulations which assumed interventions at random dates. It should be noted that the overall positive average effect in these counterfactual regressions are expected. The test was whether a random date of implementation would change the results, and none of the municipalities could start with an e-prescription system and then leave it. In this sense the structure of the random draw resembled what happened: as time passed the municipalities at some point introduced an electronic prescription system. For this reason there will be a positive average in the simulations and it is the large difference between this positive average and the actual results received when using the true dates which provide some confidence that the method in fact picks up a significant effect.

A second potentially problematic issue is that the new system changed many different variables and the current study has only estimated the overall net effect of these changes. Both direct and indirect costs changed, for both the physician and the patient. All of this contributed to the change in the prescription pattern and the data cannot isolate the separate effect of each change. For instance, the reduction in the co-payment occurred at the same time the electronic prescription system was introduced. A future line of research would be to gather new data and try to determine the importance of the various factors. For instance, to what extent the increase was caused by the direct change in financial incentives (i.e. no co-payment for the patient) versus the reduced indirect cost associated with the increased ease of asking for and approving prescriptions (for both the patient and the physician). Although it would be useful to distinguish between the strength of the different changes, the overall net effect is still important. Often a change in the prescription system will logically lead to other changes. For instance, the change in co-payment was linked to the introduction of the electronic system because it was difficult to collect co-payments when the patient did not have to go to the office to get the prescription. In this sense the changes are connected and can be evaluated as a single and logically interrelated reform package.

A third potential problem is that administrative reforms may also change reporting habits and procedures. With an electronic system, it is less likely that a prescription will not be counted and it is easier to split prescriptions into several small parts. It is difficult to determine the extent of such reporting changes from the aggregate data, but the change in overall prescriptions is interesting even if it to some extent reflect reporting changes as opposed to real changes in prescription behaviour. For policy makers deciding whether to implement these systems, the increased reimbursement costs associated with more prescriptions are real regardless of whether the cause is an actual change in prescription behaviour or a reporting change. In practice it will be a combination and to the extent that it is possible to distinguish between these sources, this would be an interesting topic for further investigation.

## Conclusion

The introduction of an electronic prescription system with no patient co-payment, led to an increase in the number of reported prescriptions of between 6.1 and 8.0 %. Although the number of prescriptions increased, the dose per prescription decreases so the new system did not significantly increase the overall consumption of drugs as measured by average daily dose. Finally, the new system did not decrease the number of office or phone consultations reimbursed to physicians.
